# Enhanced Transcription of Human Endogenous Retroviruses and TRIM28 Downregulation in Patients with Inflammatory Bowel Disease

**DOI:** 10.3390/v16101570

**Published:** 2024-10-05

**Authors:** Pier-Angelo Tovo, Davide Giuseppe Ribaldone, Ilaria Galliano, Gian Paolo Caviglia, Maddalena Dini, Valentina Veglio, Cristina Calvi, Paola Montanari, Demis Pitoni, Simone Frara, Elisa Tribocco, Anxhela Poshnjari, Massimiliano Bergallo

**Affiliations:** 1 Department of Public Health and Pediatric Sciences, University of Turin, Piazza Polonia 94, 10126 Turin, Italy; pierangelo.tovo@unito.it; 2 Department of Medical Sciences, Division of Gastroenterology, University of Turin, 10123 Turin, Italy; davidegiuseppe.ribaldone@unito.it (D.G.R.); gianpaolo.caviglia@unito.it (G.P.C.); valentina.veglio@edu.unito.it (V.V.); demis.pitoni@unito.it (D.P.); docfrara@gmail.com (S.F.); elisa.tribocco@edu.unito.it (E.T.); anxhelaposhnjari@gmail.com (A.P.); 3 Pediatric Laboratory, Department of Public Health and Pediatric Sciences, University of Turin, Regina Margherita Children’s Hospital, Piazza Polonia 94, 10126 Turin, Italy; maddalena.dini@unito.it (M.D.); cristina.calvi@unito.it (C.C.); paola.montanari@unito.it (P.M.); massimiliano.bergallo@unito.it (M.B.)

**Keywords:** Crohn’s disease, ulcerative colitis, IBD, human endogenous retroviruses, TRIM28, SETDB1, pathogenesis

## Abstract

Inflammatory bowel disease (IBD) includes patients affected by Crohn’s disease or ulcerative colitis. IBD is thought to be a chronic immune-mediated disease, but its origin remains elusive, and this limits new therapeutic approaches. Human endogenous retroviruses (HERVs) originate from ancestral infections and represent 8% of the human genome. HERVs are no longer infectious, but some retroviral sequences can be activated, and their aberrant expressions have been implicated in inflammatory and autoimmune disorders. HERV transcription is regulated by TRIM28 and SETDB1, which are also directly involved in epigenetic processes and modulation of the immune response. Using a PCR real-time Taqman amplification assay, we assessed, for the first time, the transcription levels of pol genes of HERV-H, -K, and -W families of env genes of syncytin 1 (SYN1), SYN2, and HERV-W, as well as of TRIM28 and SETDB1 in the whole blood of 48 patients with Crohn’s disease (CD), 20 with ulcerative colitis (UC), and in healthy controls (HC) of comparable age. The transcriptional levels of HERV-H-pol (*p* = 0.0003) and HERV-K-pol (*p* = 0.001) were significantly higher in IBD patients compared with HC, with no differences between patients with CD and UC. No significant differences were found for the remaining HERVs between IBD patients and HC. The transcript levels of TRIM28 were significantly downregulated in IBD patients (*p* < 0.001), without differences between CD and UC, while the SETDB1 levels were preserved. The enhanced transcription of HERV-H-pol and HERV-K-pol, as well as the impaired activation of TRIM28, were not influenced by clinical disease activity and type of treatment. The overexpression of HERVs and impaired transcription of TRIM28 in patients affected by CD or UC suggest that they might be the main actors in the pathophysiology of IBD, opening the way to innovative targeted interventions.

## 1. Introduction

Inflammatory bowel disease (IBD) is a major cause of chronic disability. It is considered an immune-mediated disease, but its pathogenesis remains poorly understood and this limits the identification of novel therapeutic measures [1,2]. The contribution of hereditable genetic factors is increasingly recognized [3,4,5]. Epigenetics is an emerging element through which environmental factors can modulate gene expressions without changing their fundamental structure. An accumulating body of literature highlights that epigenetic factors are highly involved in the development Crohn’s disease (CD) and ulcerative colitis (UC) [3,4,6,7,8]. Recent studies have also shown that the gut microbiota plays a pivotal role in maintaining gut homeostasis, and regulating local and systemic immune responses, while its imbalance may give rise to microbial metabolites able to trigger epigenetic variations in IBD patients [9,10]. Toll-like receptors (TLRs) participate in the immune response to microbial components. They contribute to preserving the intestinal epithelial barrier that can be compromised by dysregulated TLR signaling, allowing microbes to penetrate and trigger inflammatory responses in IBD [11].

Human endogenous retroviruses (HERVs) originate from the ancient infections of germinal cells of primates millions of years ago. Due to evolutionary mutations, they have lost the ability to produce infectious particles. However, HERVs still retain their retroviral structure, which consists of three principal genes: group-associated antigens (gag), polymerase (pol), and envelope (env), flanked by two regulatory long terminal repeats (LTRs) [12]. Most HERVs are inactive, but some elements are transcribed and a few encode proteins. Increasing evidence documents the essential role of HERVs during the intrauterine life. For instance, two envelope proteins, referred to as syncytin 1 (SYN1) [13] and syncytin 2 (SYN2) [14], contribute to the placental syncytiotrophoblast formation and the materno-foetal tolerance through their vigorous immune-suppressive effects [15]. Postnatally, the physiologic actions of HERVs are unclear: while activated retroviruses can act as the promoters or enhancers of cellular genes [16,17], their RNAs can be retrotranscribed and reintegrated into the DNA or, being recognized as non-self by TLRs, induce innate and specific immune responses [17,18,19]. In fact, HERVs have been studied and proposed in the pathophysiology of many inflammatory immune-mediated diseases [20,21,22,23,24]. HERVs also have a mutual influence on microbiota, and their enhanced expression promotes microbiota-mediated gut inflammation [25].

HERV transcription is influenced by environmental factors through epigenetic mechanisms, such as DNA methylation and histone modification, leading to heterochromatin silencing. The tripartite motif-containing 28 (TRIM28, also known as KAP1 or TIF1-b), functions as a nuclear corepressor for Krüppel-associated box domain zink finger proteins (KRAB-ZFPs), which are the largest family of transcriptional regulators in the human genome [26]. SET domain bifurcated histone lysine methyltransferase 1 (SETDB1) is a methyltransferase highly specific for the lysine 9 residue of histone H3 [27]. It has multifaced biological properties, such as T cell development, intestinal epithelial cell differentiation, and the prevention of gut inflammation [28,29,30]. Both TRIM28 and SETBD1 are specific tags for the epigenetic transcriptional repression of HERVs [31,32,33]. Furthermore, they regulate the transactivation of thousands of cellular genes [34,35] and are directly involved in epigenetic processes [32], including the modulation of innate and adaptive immune responses [36,37]. 

Despite the aforementioned elements supporting the potential role of HERVs, TRIM28, and SETDB1 in inducing and/or maintaining immune-mediated inflammatory disorders, no studies investigated their expressions in patients affected by IBD except for one study on syncytin expression in intestinal biopsies from CD patients [38]. Therefore, the aims of our research were to evaluate the transcriptional levels of pol genes of HERV-H, HERV-K, and HERV-W, the three retroviral families most widely studied [12,39]; env genes of SYN1, SYN2, and HERV-W [21,40]; as well as TRIM28 and SETDB1 in the whole blood from adults affected by CD or UC, and in healthy controls (HC) of comparable age.

## 2. Material and Methods

### 2.1. Study Populations

The diagnosis of CD and UC was based on clinical, radiological, and endoscopic findings according to the European guidelines [41]. Patients’ peripheral blood samples were collected during routine laboratory checks. Healthy adult volunteers of comparable age were the control group. 

### 2.2. Sample Storage

Of the whole blood, 200 μL was added to 800 µL of the RNApro solution (Biomole, Turin, Italy) in a 1.5 mL Eppendorf tube and resuspended by vortexing; the samples were stored at −80 °C [42].

### 2.3. Total RNA Extraction 

Total RNA was extracted using the Maxwell automated extractor in combination with the RNA Blood Kit (Promega, Madison, WI, USA). This kit includes DNase treatment during the extraction process. To ensure no genomic DNA contamination, RNA extracts were directly amplified without reverse transcription to validate the extraction protocol. RNA concentration and purity were evaluated by UV spectroscopy, measuring absorbance at 260 and 280 nm SimpliNano spectrophotometer (Biochrom US, Holliston, MA, USA). The RNAs were stored at −80 °C until use.

### 2.4. Reverse Transcription 

Four hundred nanograms of total RNA were reverse-transcribed in a 20 μL reaction mixture containing 2 μL of a 10× buffer, 4.8 μL of 25 mM MgCl2, 2 μL ImProm-II reverse transcriptase (Promega), 1 μL of RNase inhibitor (20 U/L), 0.4 μL of 250 μM random hexamers (Promega), 2 μL of 100 mM dNTP mix (Promega), and nuclease-free water. The reverse transcription reaction was performed in a GeneAmp PCR system 9700 Thermal Cycle (Applied Biosystems, Foster City, CA, USA) under the following conditions: 5 min at 25 °C, 60 min at 42 °C, followed by 15 min at 70 °C for enzyme inactivation. The cDNAs were stored at −20 °C until use.

### 2.5. Transcription Levels of pol Genes of HERV-H, -K, -W; env Genes of SYN1, SYN2, and HERV-W; as Well as TRIM8/SETDB1 by a Real-Time PCR Assay

The relative expression levels of pol genes from HERV-H, HERV-K, and HERV-W; env genes from SYN1, SYN2, and HERV-W; along with TRIM28/SETDB1 were measured, as previously detailed, using the primers and probes listed in Supplementary Table S1 [22,23,24]. Briefly, 40 ng of cDNA was amplified in a 20 μL reaction containing 2.5 U goTaQ MaterMix (Promega), 1.25 mmol/L MgCl2, 500 nmol of specific primers, and 200 nmol of specific probes. All the amplifications were performed in a 96-well plate under the following conditions: initial denaturation at 95 °C for 10 min, followed by 45 cycles at 95 °C for 15 s and at 60 °C for 1 min. Each sample was analyzed in triplicate. The relative expression of target gene transcripts was carried out according to the 2^−ΔΔCt^ method [43]. GAPDH was chosen as the reference gene, due to its consistent expression in human leukocytes and its proven efficiency and excellent reproducibility [44], as previously observed in our studies [22,23,24]. Briefly, after normalization of the PCR result of each target gene with the housekeeping gene, the method includes additional calibration of this value with the median expression of the same gene emerging in a pool of healthy controls. The results of the 2^−ΔΔCt^ method show variations in target gene transcripts relative to the standard set of controls. As documented in this and other investigations, the expression of HERVs, TRIM28, and SETDB1 varies greatly among healthy individuals. The reason is unknown and in the literature, no definition of normal values or normal threshold has been proposed; consequently the analyses to assess potential significant differences between IBD patients and HC were performed by comparing all their values. Since we measured Ct for every target in all samples, we argue that our methods were suitable for HERV and TRIM28/SETDB1 quantifications. All analyses were carried out in a laboratory of biosafety level 2 (BSL-2) according to the Office of Science Policy and WHO guidelines [45,46].

### 2.6. Statistical Analysis

A one-way ANOVA test was employed to compare the transcriptional levels of each target gene among patients with CD, UC, and HC. Since the Shapiro–Wilk test to evaluate the distribution of data of every group of subjects demonstrated non-normally distributed continuous variables, the Mann–Whitney test was used to assess differences in the relative transcription levels of pol genes from HERV-H, HERV-K, and HERV-W; env genes from SYN1, SYN2, and HERV-W; and TRIM28 and SETDB1 genes between two groups of subjects. Statistical analyses were performed using the Prism 7 software (GraphPad Software); *p* < 0.05 was considered statistically significant.

## 3. Results

### 3.1. Study Population

A total of 68 patients affected by IBD were studied: 48 with CD (Group A1) and 20 with UC (Group A2). The control subjects grouped healthy volunteers, selected without selection criteria. They were divided into two groups according to the tests performed: Group B1 included 104 subjects whose blood samples were also used for other studies and whose stored cDNAs were sufficient to be tested for pol genes of HERV-H, HERV-K, and HERV-W; while Group B2 included 81 healthy volunteers tested for the other targets. The control subjects were selected with the gender and age comparable to the patients. The median ages of IBD patients were comparable to those of HC: A1 vs. B1 *p* = 0.1376, A2 vs. B2 *p* = 0.1950. The characteristics of the patients and control subjects are detailed in Table 1.

### 3.2. Transcription Levels of HERV-H-pol, HERV-K-pol, and HERV-W-pol in the Whole Blood of Patients with Crohn’s Disease, Ulcerative Colitis, and HC

**Figure 1:**
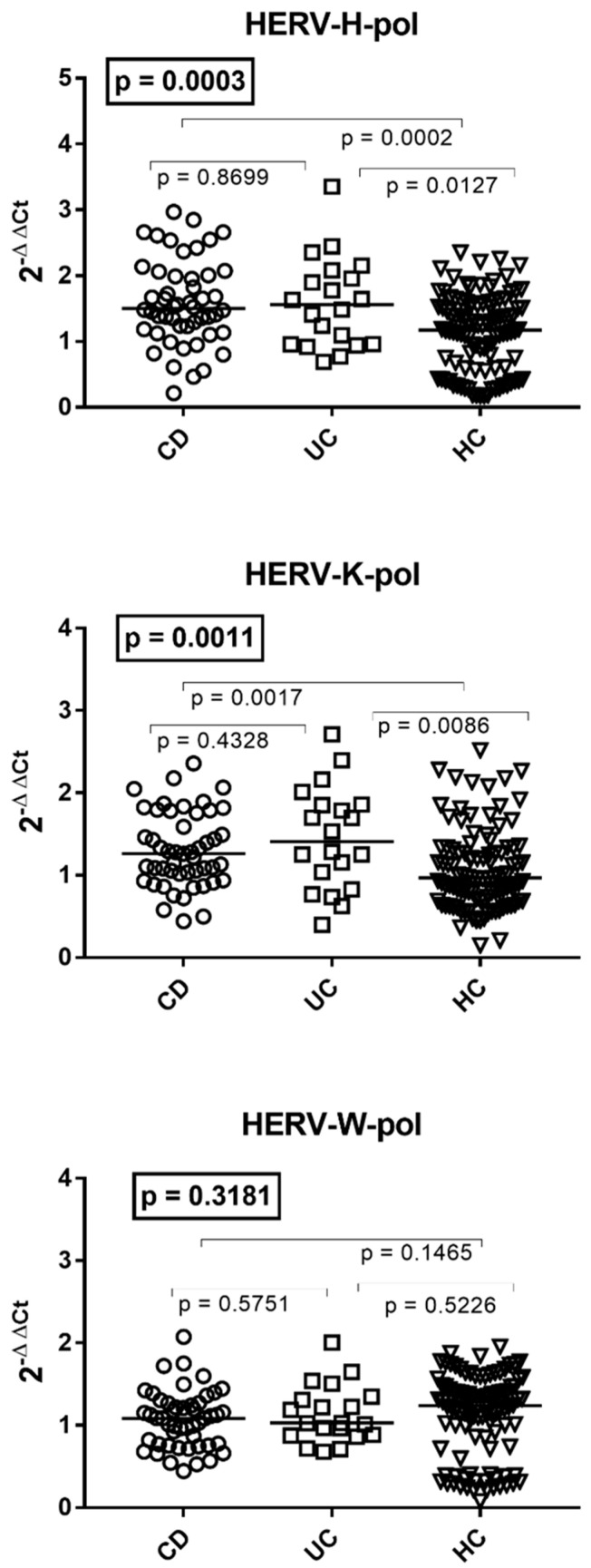
Transcription levels of the pol genes of HERV-H, HERV-K, and HERV-W in the whole blood from 48 patients with Crohn’s disease, 20 with ulcerative colitis (UC), and 104 healthy controls (HC). 2^−ΔΔCt^ = relative expression according to the 2^−ΔΔCt^ method. Circles, squares, and triangles show the median of three individual measurements; horizontal lines represent the median values. The boxed *p*-values represent the result of a one-way ANOVA test, while the other *p*-values represent the result of the Mann–Whitney test.

The transcriptional levels of HERV-H-pol and HERV-K-pol were significantly higher in patients with CD or UC than in HC of comparable age, while no differences were found between the two subpopulations of IBD patients (Figure 1). The mRNA levels of HERV-W-pol were comparable between subjects with CD, UC, and HC. The medians and IQR 25–75% were as follows: HERV-H-pol CD 1.53, 1.22–2.02; UC 1.56, 0.96–1.99, HC 1.24, 0.63–1.63; HERV-K-pol CD 1.27, 1.00–1.76; UC 1.41, 0.99–1.85; HC 0.97, 0.71–1.34; HERV-W-pol CD 1.09, 0.78–1.27; UC 1.03, 0.88–1.32; HC 1.32, 0.86–1.59 (Figure 1). 

### 3.3. Transcription Levels of the env Genes of Syncytin 1, Syncytin 2, and HERV-W in the Whole Blood of Patients with Crohn’s Disease, Ulcerative Colitis, and HC

**Figure 2:**
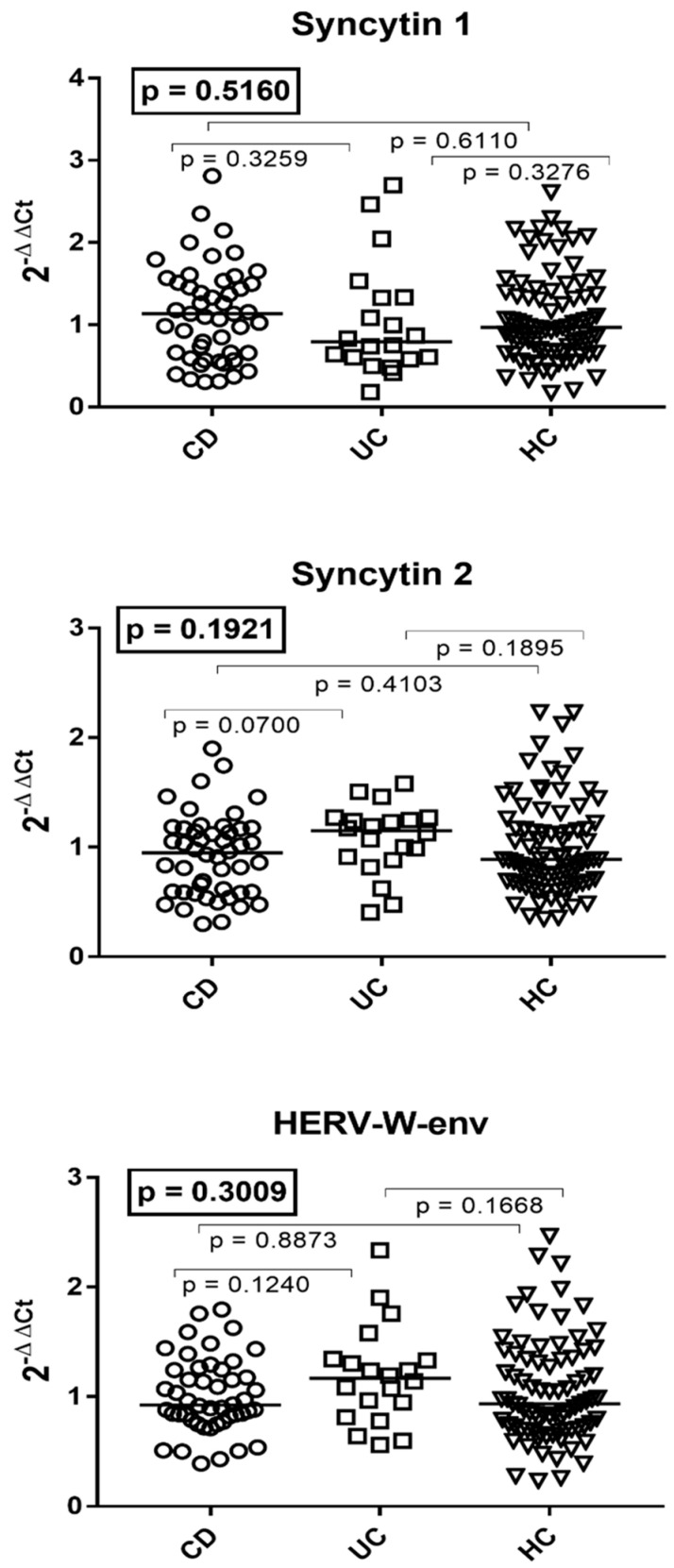
Transcription levels of the env genes of syncytin 1, syncytin 2, and HERV-W in the whole blood from 48 patients with Crohn’s disease (CD), 20 with ulcerative colitis (UC), and 81 healthy controls (HC). 2^−ΔΔCt^ = relative expression according to the 2^−ΔΔCt^ method. Circles, squares, and triangles show the median of three individual measurements; horizontal lines represent the median values. The boxed *p*-values represent the result of a one-way ANOVA test, while other *p*/values represent the result of the Mann–Whitney test.

The median values of the env genes of SYN1, SYN2, and HERV-W were similar in patients with CD, UC, and HC of comparable age (Figure 2). The medians and IQR 25–75% were as follows: syncytin 1 CD 1.14, 0.66–1.52; UC 0.79, 0.6–1.33; HC 1.02, 0.70–1.48; syncytin 2 CD 0.95, 0.59–1.17; UC 1.15, 0.91–1.53; HC 0.93, 0.70–1.38. HERV-W CD 0.93, 0.81–1.25; UC 1.17, 0.9–1.34; HC 0.98, 0.73–1.44 (Figure 2).

### 3.4. Transcription Levels of TRIM28 and SETDB1 in Patients with Crohn’s Disease, Ulcerative Colitis, and HC

**Figure 3:**
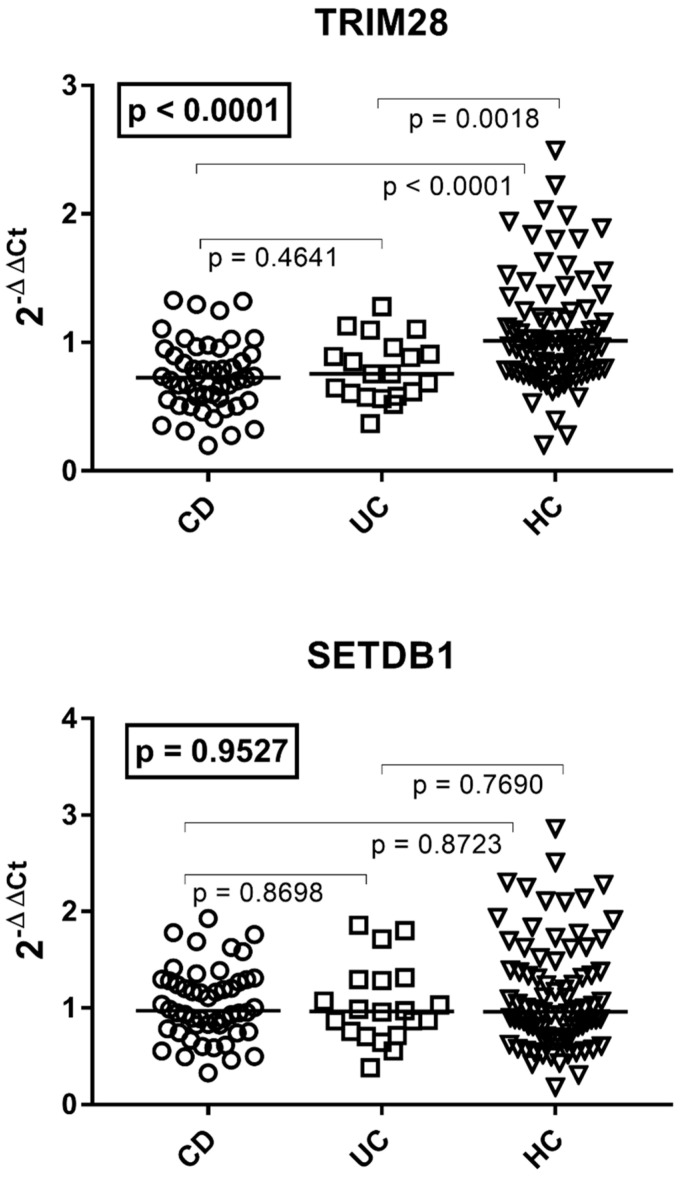
Transcription levels of TRIM28 and SETDB1 in the whole blood from 48 patients with Crohn’s disease (CD), 20 with ulcerative colitis (UC), and 81 healthy controls (HC). 2^−ΔΔCt^ = relative expression according to the 2^−ΔΔCt^ method. Circles, squares, and triangles show the median of three individual measurements; horizontal lines represent the median values. The boxed *p*-values represent the result of a one-way ANOVA test, while the other *p*-values represent the result of the Mann–Whitney test.

As reported in Figure 3, the median transcript levels of TRIM28 were significantly lower in patients with CD or UC than in HC of comparable age, without a significant difference between the two groups of patients. The transcription levels of SETDB1 were comparable in the three groups of subjects. The medians and IQR 25–75% were as follows: TRIM28 CD 0.73, 0.56–0.92; UC 0.76, 0.60–0.92; HC 1.01, 0.79–1.25; SETDB1 CD 1.00, 0.81–1.29; UC 0.96, 0.75–1.29; HC 1.00, 0.74–1.40 (Figure 3).

### 3.5. Expressions of HERVs, TRIM28, and SETDB1 in IBD Patients According to Disease Activity

No significant differences emerged for HERVs, TRIM28, and SETDB1 between patients in remission (R) compared with those with active disease (including mild, moderate, and severe clinical disease activity, MMS) (Supplementary Figure S1). The medians and IQR 25–75% were as follows: HERV-H-pol R 1.58, 1.12–2.10; MMS 1.49, 1.22–1.91; HERV-K-pol R 1.29, 1.07–1.78; MMS 1.13, 0.90–1.79; HERV-W-pol R 1.11, 0.88–1.35; MMS 0.98, 0.81–1.24; syncytin 1 R 1.12, 0.65–1.50; MMS 0.98, 0.60–1.43; syncytin 2 R 1.03, 0.68–1.29; MMS 1.00, 0.59–1.17; HERV-W-env R 1.11, 0.84–1.27; MMS 0.95, 0.75–1.18; TRIM28 R 0.76, 0.58–0.92; MMS 0.72, 0.58–0.90; SETDB1 R 0.97, 0.86–1.30; MMS 0.98, 0.70–1.27 (Supplementary Figure S1).

### 3.6. Transcription Levels of HERVs, TRIM28, and SETDB1 in IBD Patients According to Mesalazine Treatment

The median mRNA levels of HERVs, TRIM28, and SETDB1 were comparable between patients treated with mesalazine (Mes) and those without mesalazine (No Mes) (Supplementary Figure S2). The medians and IQR 25–75% were as follows: HERV-H-pol Mes 1.48, 1.16–2.07; No Mes 1.64, 1.09–1.82; HERV-K-pol Mes 1.26, 1.03–1.79; No Mes 1.29, 0.91–1.70; HERV-W-pol Mes 1.09, 0.88–1.36; No Mes 0.97, 0.82–1.20; Syncytin 1 Mes 1.08, 0.59–1.48; No Mes 0.99, 0.66–1.51; Syncytin Mes 1.06, 0.66–1.29; No Mes 0.91, 0.58–1.05; HERV-W-env Mes 1.07, 0.85–1.33; No Mes 0.84, 0.78–1.09; TRIM28 Mes 0.76, 0.59–0.93; No Mes 0.67, 0.50–0.85; SETDB1 Mes 0.99, 0.84–1.29; No Mes 0.97, 0.71–1.28 (Supplementary Figure S2).

### 3.7. Transcription Levels of HERVs, TRIM28, and SETDB1 in IBD Patients According to Steroid Treatment

The median transcription levels of HERVs, TRIM28, and SETDB1 were comparable between patients treated with steroids and those untreated (Supplementary Figure S3). The medians and IQR 25–75% were as follows: HERV-H-pol steroids 1.56, 1.24–1.96; no steroids 1.48, 1.00–2.05; HERV-K-pol steroids 1.29, 1.08–1.73; no steroids 1.27, 0.87–1.79; HERV-W-pol steroids 1.12, 0.89–1.28; no steroids 1.06, 0.86–1.27; syncytin 1 steroids 1.23, 0.68–1.53; no steroids 0.99, 0.56–1.36; syncytin 2 steroids 0.89, 0.63–1.18; no steroids 1.06, 0.64–1.19; HERV-W-env steroids 1.00, 0.84–1.26; no steroids 1.02, 0.80–1.28; TRIM28 steroids 0.68, 0.57–0.83; no steroids 0.75, 0.58–0.96; SETDB1 steroids 1.01, 0.78–1.26; no steroids 0.96, 0.80–1.29 (Supplementary Figure S3).

### 3.8. Expressions of HERVs, TRIM28, and SETDB1 in IBD Patients with and without Anti-Tumor Necrosis Factor (TNF) Treatment

The expressions of HERVs, TRIM28, and SETB1 were comparable between IBD patients treated (anti-TNF POS) or untreated with anti-TNF (anti-TNF NEG) (Supplementary Figure S4). The medians and IQR 25–75% were as follows: HERV-H-pol anti-TNF POS 1.75, 1.27–2.31; anti-TNF NEG 1.48, 1.13–1.94; HERV-K-pol POS 1.31, 1.08–1.86; NEG 1.27, 0.93–1.70; HERV-W-pol anti-TNF POS 1.15, 0.97–1.26; anti-TNF NEG 1.03, 0.83–1.33; Syncytin 1 anti-TNF POS 0.97, 0.62–1.33; anti-TNF NEG 1.05, 0.62–1.53; Syncytin 2 anti-TNF POS 0.99, 0.84–1.18; anti-TNF NEG 1.03, 0.60–1.20; HERV-W-env anti-TNF POS 1.03, 0.84–1.32; anti-TNF NEG 1.00, 0.79–1.25; TRIM28 anti-TNF POS 0.87, 0.58–0.95; anti-TNF NEG 0.73, 0.58–0.88; SETDB1 anti-TNF POS 1.20, 0.94–1.30; anti-TNF NEG 0.95, 0.75–1.23 (Supplementary Figure S4).

## 4. Discussion

Our results document, for the first time, that patients affected by IBD exhibit significantly higher transcriptional levels of HERV-H-pol and HERV-K-pol in peripheral blood compared with healthy controls of similar age. The RNA levels of HERV-W-pol and the env genes of SYN1, SYN2, and HERV-W were comparable between patients and the control group. No significant difference in the expression of every HERV was observed between patients with CD and those with UC. 

The HERV-H is the largest family of retroviruses in the human genome, while the HERV-K is the most recent component, present only in humans. The underlying biological mechanisms responsible for their aberrant expression in patients with IBD and their clinical significance remain to be elucidated. An extensive body of literature highlights that TRIM28 is implicated in maintaining endogenous retroviruses in a silent state [31,32]. The impaired transcriptional levels of TRIM28 in our patients may thus account for the enhanced expression of some HERV elements. SUMOylated TRIM8 is a scaffold protein recruiting SETDB1 to interact with KRAB-ZFPs to repress retroelements [32,49]. SETDB1 expression was normally represented in whole blood of our patients, in line with the preserved mucosal SETDB1 transcript levels in most patients with IBD [50], though others found a relative deficiency [28]. Rare missense variants of SETDB1 are over-represented in IBD and these have been suggested to participate in its pathogenesis [29]. It cannot be overlooked that functional interactions between TRIM28/SETDB1 and single HERVs may originate from post-translational events between the encoded proteins, while we assessed only their transcriptional landscape. Finally, in addition to the TRIM28/SETDB1/KRAB-ZFP complex, presumably a number of other genes are implicated in the control of HERV silencing; therefore, they could contribute to the abnormal expression of some retroviral elements in our patients.

Growing data document the increased release of pro-inflammatory cytokines in subjects with IBD [2]. Inflammatory cytokines give rise to the proteasome-driven activation of the NF-kB signaling pathway. The active isoform of NF-kB, after passage into the nucleus, binds to specific retroviral sequences that, along with inflammatory cytokines, lead to their enhanced transactivation [51]. It must be underlined that HERVs can, in turn, evoke robust inflammatory and immune responses [12,39] and exert several pathogenetic actions. It is worth mentioning that, recently, the gene enhancer ETS2, located in the non-coding desert zone of chr21q22, has been shown to play a major role in IBD, directing macrophage inflammation [5]. Interestingly, HERVs can be the promoters and enhancers of cellular genes [16,17]. HERV RNAs can be retrotranscribed and reintegrated into the DNA, causing possible mutations. The recognition of HERV RNAs by nucleic acid-sensing TLRs may lead to activation of the inflammasome [17,18]; for instance, HERV-K stimulates the NF-kB pathway through TLR8 [19]. Additionally, HERV antigens can trigger targeted responses, including specific and/or cross-reactive antibodies with tissue epitopes [52,53,54,55]. The final result may be a vicious circle leading to chronic inflammatory and immune reactions. In this context, it must be pointed out that enhanced HERV expressions have been documented in several autoimmune diseases [21,22,23,39]. 

There are mutual interactions between gut microbiota and endogenous retroviruses [25]. Germ-free mice lose intestinal expression of many retroviruses, while exposure to bacteria and their products can stimulate retroviral transcription [56]. The dysbiosis of the gut microbiota present in IBD patients might thus contribute to their enhanced HERV expression. 

In contrast to our findings, the downregulation of syncytins has been reported in intestinal biopsies of patients affected by CD compared to those of normal subjects [38]. This discrepancy might be due to the sample size or the effect of certain treatments. On the other hand, the intestinal mucosa shows a regular expression of HERVs [57]. Diffuse staining of normal crypt cells was seen using an anti-HERV monoclonal antibody, whereas no background staining was observed in endothelial and infiltrating immune cells [58]. In CD, patients’ intestinal cells and stem cells [59], which are characterized by the highest levels of HERV expression [31,32], are reduced and replaced by the intense leukocyte infiltrate. These histologic changes might account for the defective syncytin expression in biopsies from CD patients.

There is wide consensus that abnormal epigenetic processes triggered by environmental factors contribute to the development of IBD [3,6,7,8], but the precise responsible pathways remain unknown. TRIM28 is highly implicated in the regulation of epigenetic mechanisms; its impaired transcription in our patients is the first specific molecular alteration documented in subjects affected by IBD. TRIM28 influences the differentiation, expansion and activation of T cells. Through a complex with KRAB-ZFPs and Foxp3, it modulates Treg suppressor activity [50,60]. Furthermore, TRIM28 represses the expression of inflammatory genes, while its deficiency increases the expansion of DCs and enhanced T cell priming toward inflammatory effector T cells via HERV activation [50]. Therefore, the reduced transcript levels of TRIM28 in IBD patients may contribute to their deficiency in T regulatory cells and the expansion of reactive T lymphocytes, ultimately giving rise to derailed local and systemic immune responses, with consequent autoimmune phenomena in the intestinal tract [36]. 

The expression levels of every HERV and TRIM28/SETDB1 were not associated with disease activity or treatment with mesalazine, steroids, or anti-TNF. These findings suggest that the variables here taken into consideration are independent from the disease state and are not influenced by these therapies. The prevalent number of patients in remission or with mild disturbances does not allow, however, for definitively ruling out the potential relationship of some variables with moderate/severe forms of disease in untreated or unresponsive patients. In contrast, all our patients were treated with one or more drugs, which played the main role in keeping the disease under control. 

The high incidence and prevalence of IBD has reached a plateau in recent years in the Western world, whereas the disease burden is continuously increasing in newly industrialized Asian Pacific countries [61,62]. The reason(s) is ill-defined. Changes in diet and the addition of food additives [63], Western lifestyle, smoking, urban environment, and composition of enteric microbiome have been associated with an increased frequency of IBD in a genetically susceptible host [64]. Diet [65], smoking [66], and dysbiosis in commensal microorganisms [67] can act as epigenetic elements promoting CD and UC [68,69]. In addition, cigarette smoking [70], pollution [71], nutritional changes linked to lifestyle [72], and gut microbiota [56] are implicated in retrovirus expression too. Therefore, environmental factors, thought to play an essential role in the development of IBD, could exert their effects via HERV- and/or TRIM28-driven changes in specific biologic pathways. 

Our findings raise further intriguing questions. Are the overexpression of HERVs and the downregulation of TRIM28 the biomarkers of IBD? Several anti-HERV therapeutic measures might be adopted, such as specific anti-RNAs, monoclonal antibodies, cytotoxic T lymphocytes against HERV antigens, and antiretroviral treatments [73,74,75]. Antiretroviral drugs inhibited both HIV viral burden and HERV expression in HIV+ subjects [76,77]. Combined antiretroviral treatment in patients with amyotrophic lateral sclerosis showed a better disease course in those with positive antiviral findings [15]. A novel anti-HIV product [78] induced positive effects in a phased II study in patients with UC [79]. Antiretroviral drugs inhibit proteasome activity [80], with a consequent blocking of NF-kB-driven HERV transcription [51]. Epigenetic variations observed in IBD are increasingly indicated as potential therapeutic targets [6,69,81], in particular the TRIM protein family [82].

In conclusion, our results suggest that HERVs and TRIM28 might be implicated in the pathophysiology of CD and UC, and may provide insights toward the development of innovative therapeutic interventions.

## Figures and Tables

**Table 1:** Demographics and clinical characteristics of patients with Crohn’s disease (CD) or ulcerative colitis (UC), and healthy volunteers (Groups B1 and B2).

	Group A1 (CD) *n* = 48	Group A2 (UC) *n* = 20	Group B1 (HC) *n* = 104	Group B2 (HC) *n* = 81
Median age (IQR)	46.3 years (33.3–56.2)	55.9 years (42.8–65.8)	41.5 years (34.7–55.4)	41.0 years (33.9–52.7)
Males *n* (%)	26 (53.1)	12 (60.0)	62 (59.6)	48 (59.3)
Duration of disease (yrs) (IQR)	8 (3–20.3)	8.5 (5–22.3)		
Resection *n* (%)	19 (39.6)	3 (15)		
Clinical disease activity *				
Remission *n* (%)	28 (58.3)	12 (60)		
Mild *n* (%)	13 (27.1)	8 (40)		
Moderate *n* (%)	6 (12.5)	-		
Severe *n* (%)	1 (2.1)	-		
Treatment				
Mesalazine *n* (%)	33 (68.8)	12 (60)		
Topic steroids *n* (%)	11 (22.9)	3 (15)		
Systemic steroids *n* (%)	6 (12.5)	3 (15)		
Anti-TNF *n* (%)	14 (29.2)	5 (25)		
Ustekinumab *n* (%)	4 (8.3)	-		
Vedolizumab *n* (%)	1 (2.1)	-		
Anti-Jak *n* (%)	1 (2.1)	1 (5)		

*n*: number; IQR: interquartile range, expressed as 25 and 75 quartile values; TNF: tumor necrosis factor. * According to HBI score in CD [47] and pMAYO score in UC [48].

## Data Availability

The data underlying this article will be shared at the aggregate/population level upon reasonable request to the corresponding author.

## Supplementary Materials

The following supporting information can be downloaded at: https://www.mdpi.com/article/10.3390/v16101570/s1, Figure S1: Expression of HERVs, TRIM28 and SETDB1 according to disease activity; Figure S2: Expression of HERVs, TRIM28 and SETDB1 with and without Mesalazine; Figure S3: Expression of HERVs, TRIM28 and SETDB1 with and without Steroids; Figure S4: Expression of HERVs, TRIM28 and SETDB1 with and without anti-TNF; Table S1: Primers and probes used to assess expression of HERVs, TRIM28 and SETDB1.
