# Supplementary material for: Enhanced Transcription of Human Endogenous Retroviruses and TRIM28 Downregulation in Patients with Inflammatory Bowel Disease
Source: Viruses. 2024 Oct 5;16(10):1570. doi: 10.3390/v16101570 (PMC11512322; doi:10.3390/v16101570)
Supplement: Supplementary file 1 [file viruses-16-01570-s001.zip › viruses-3191334-supplementary.pdf]

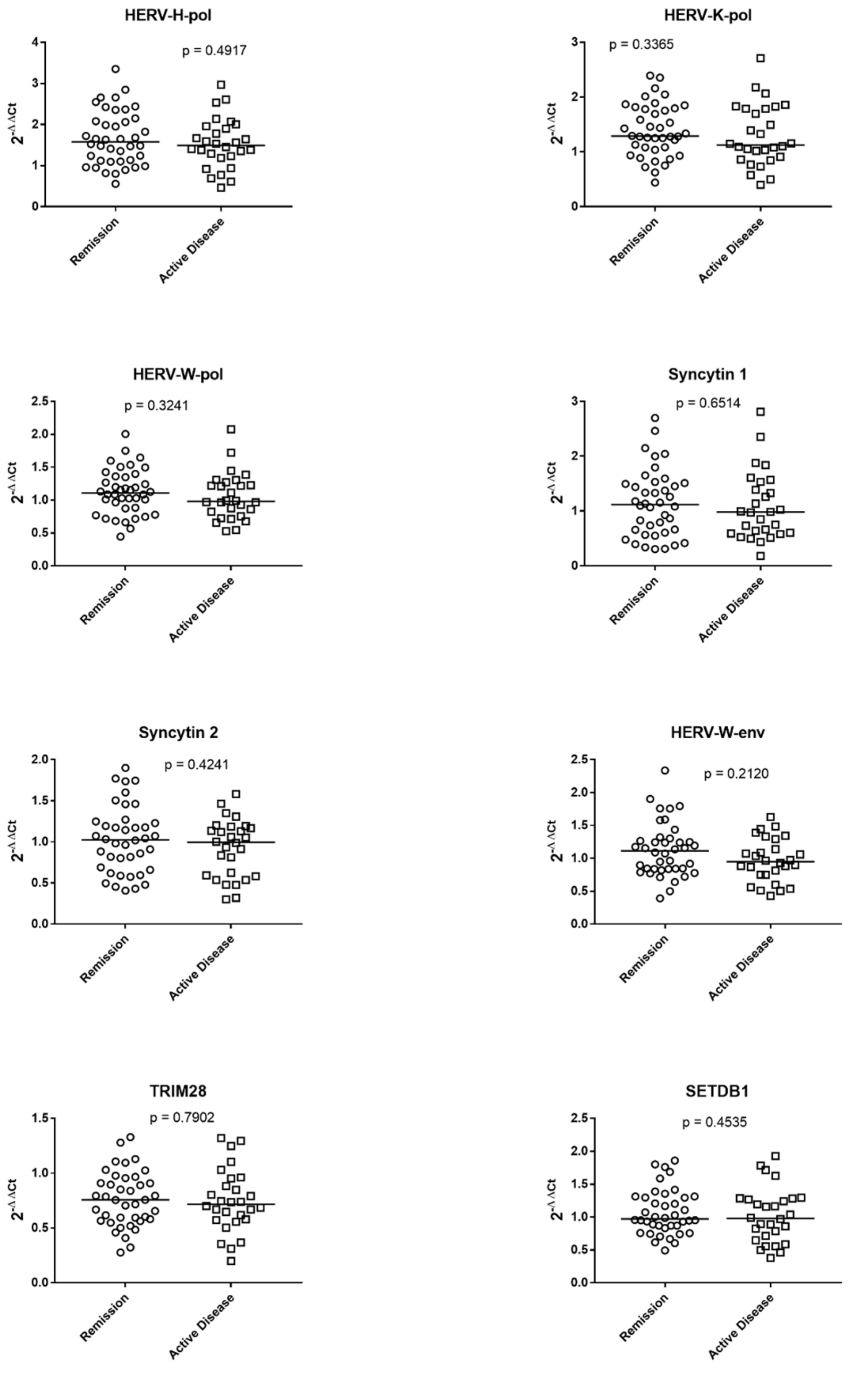

Supplementary Figure S1. Transcription levels of HERVs, TRIM28 and SETDB1 in whole blood from 40 IBD patients in remission (R) versus 28 patients with active disease (mild, moderate and severe forms). $2^{-\Delta\Delta Ct}$ = Relative Expression according to the $2^{-\Delta\Delta Ct}$ method. Circles and squares show the median of three individual measurements, horizontal lines the median values. The p values represent the result of Mann-Whitney test.

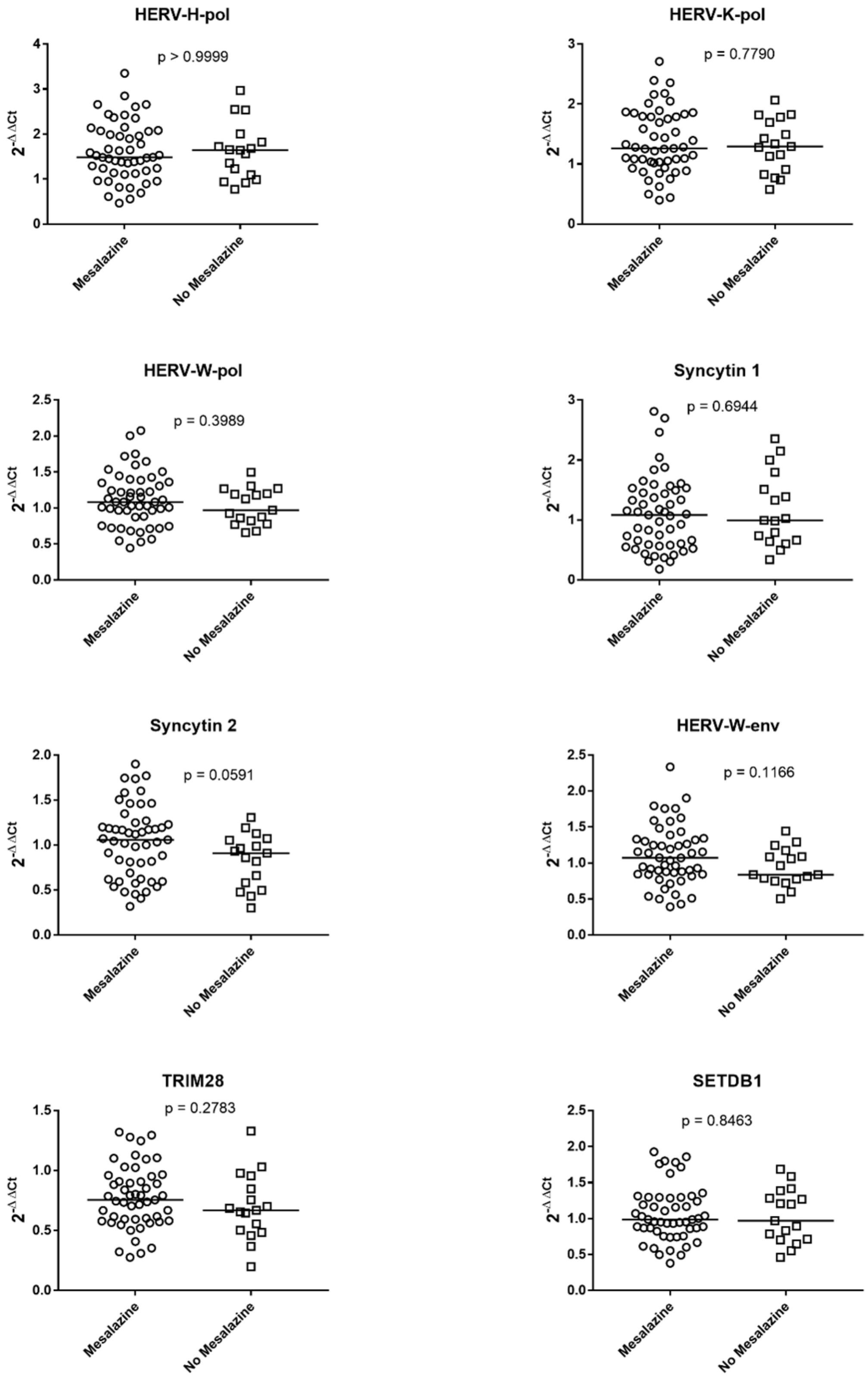

Supplementary Figure S2. Transcription levels of HERVs, TRIM28 and SETDB1 in whole blood from 51 patients treated with Mesalazine (Mes) and 17 patients without Mesalazine (No Mes). $2^{-\Delta\Delta Ct}$ = Relative Expression according to the $2^{-\Delta\Delta Ct}$ method. Circles and squares show the median of three individual measurements, horizontal lines the median values. The p values represent the result of Mann-Whitney test.

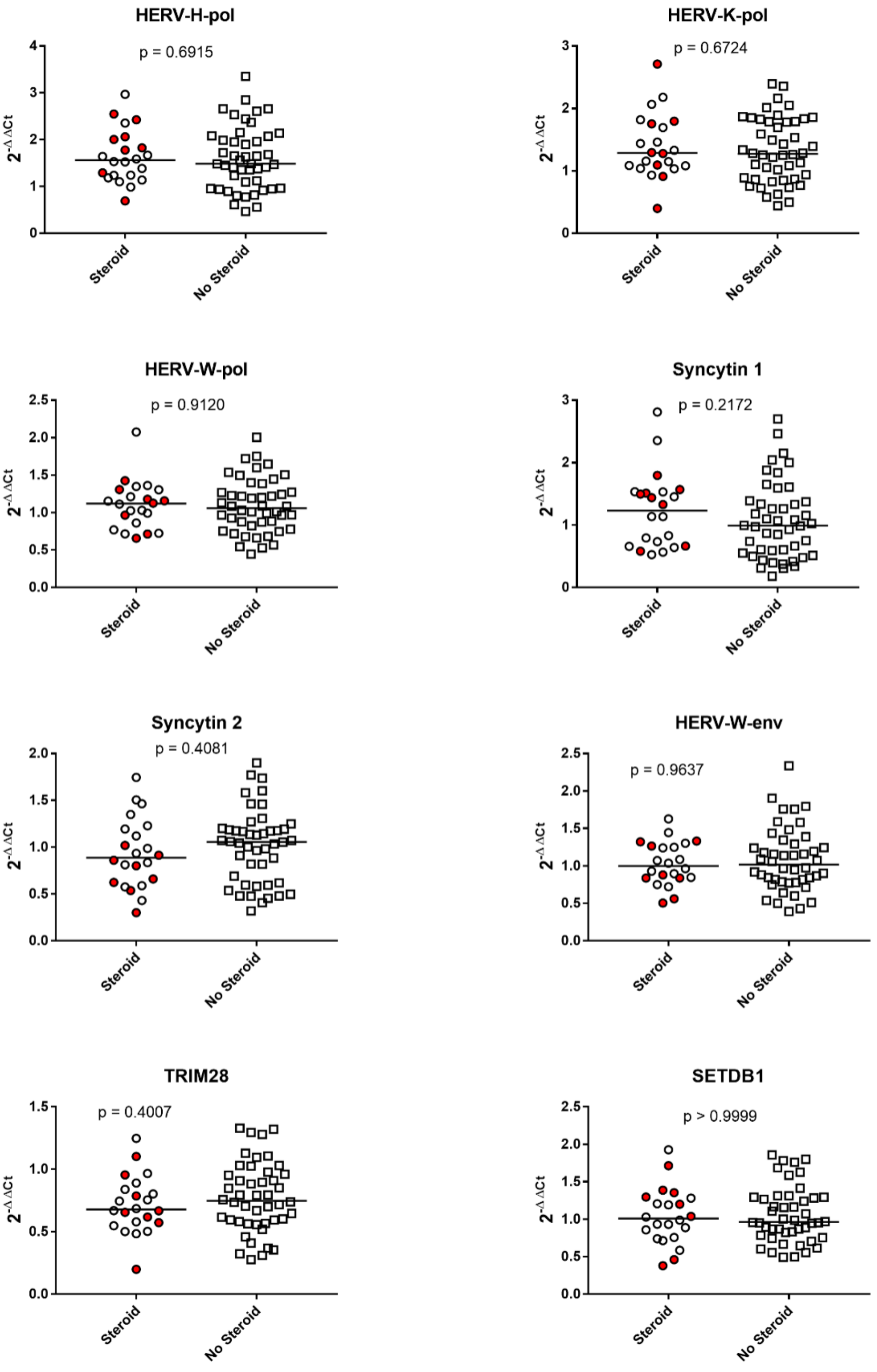

Supplementary Figure S3. Transcription levels of HERVs, TRIM28 and SETDB1 in whole blood from 22 patients treated with Steroids and 46 patients without steroids. $2^{-\Delta\Delta Ct}$ = Relative Expression according to the $2^{-\Delta\Delta Ct}$ method. Circles and squares show the median of three individual measurements, horizontal lines the median values. The p values represent the result of Mann-Whitney test. 8 patients were treated with systemic steroid (in red); the others 14 were treated with local steroid.

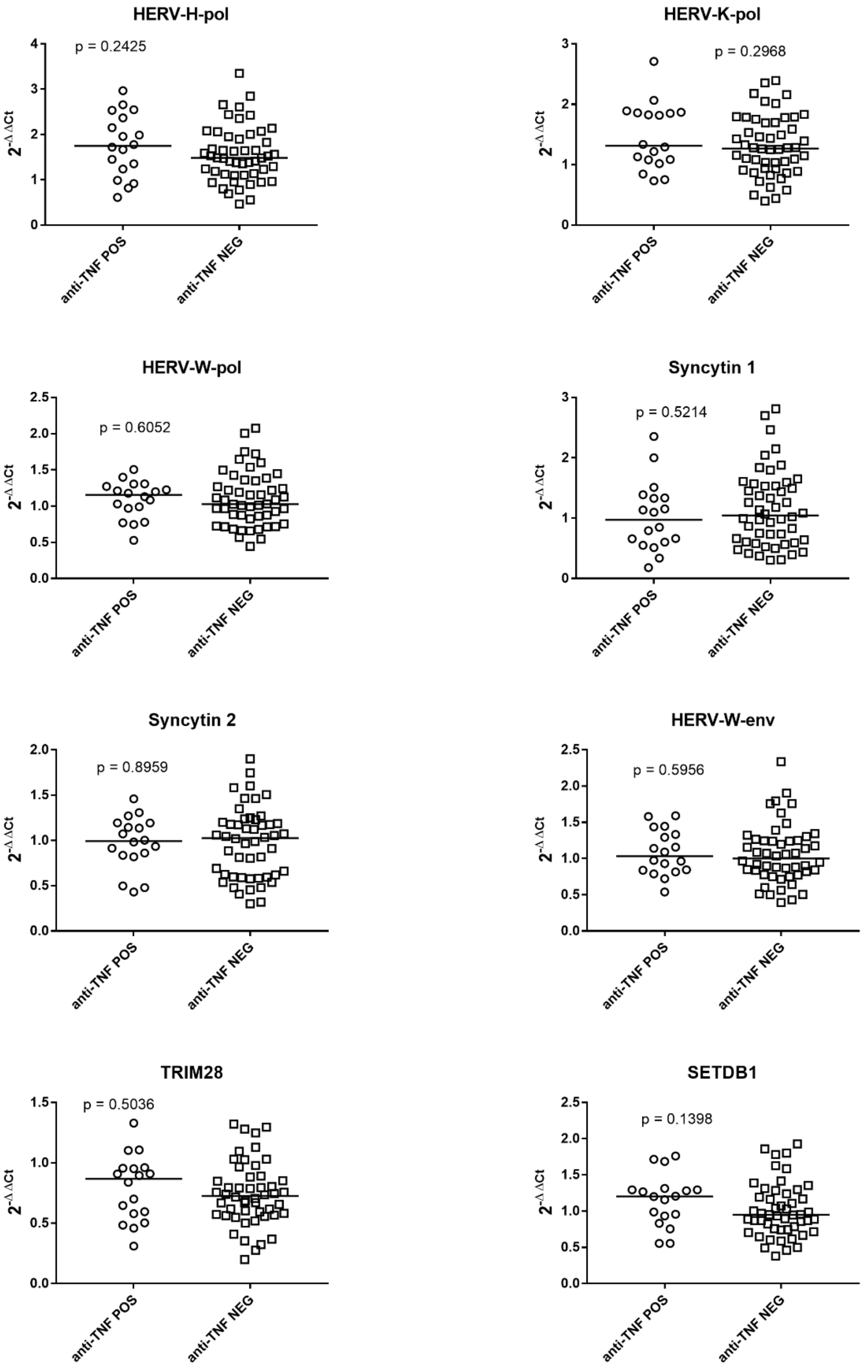

Supplementary Figure S4. Transcription levels of HERVs, TRIM28 and SETDB1 in whole blood from 18 IBD patients treated with anti-TNF (POS) and 50 patients without anti-TNF (NEG). $2^{-\Delta\Delta Ct}$ = Relative Expression according to the $2^{-\Delta\Delta Ct}$ method. Circles and squares show the median of three individual measurements, horizontal lines the median values. The p values represent the result of Mann-Whitney test.

Supplementary Table S1. Primers and probes used to assess the transcription levels of pol genes of HERV-H, HERV-K, and HERV-W, of env genes of Syncytin 1, Syncytin 2, and HERV-W, of TRIM28, SETDB1, and of GADPH.

Name	Primer/Probe	Sequence
HERV-H pol	Forward	5′-TGGACTGTGCTGCCGCAA-3′
	Reverse	5′-GAAGSTCATCAATATATTGAATAAGGTGAGA-3′
	Probe	6FAM-5′-TTCAGGGACAGCCCTCGTTACTTCAGCCAAGCTC-3′-TAMRA
HERV-K pol	Forward	5′-CCACTGTAGAGCCTCCTAAACCC-3′
	Reverse	5′-TTGGTAGCGGCCACTGATTT-3′
	Probe	6FAM-5′-CCCACACCGGTTTTTCTGTTTTCCAAGTTAA-3′-TAMRA
HERV-W pol	Forward	5′-ACMTGGAYKRTYTTRCCCCAA-3′
	Reverse	5′-GTAAATCATCCACMTAYYGAAGGAYMA-3′
	Probe	6FAM-5′-TYAGGGATAGCCCYCATCTRTTTGGYCAGGCA-3′-TAMRA
Syncytin 1 env	Forward	5′-ACTTTGTCTCTTCCAGAATCG-3′
	Reverse	5′-GCGGTAGATCTTAGTCTTGG-3′
	Probe	6FAM-5′-TGCATCTTGGGCTCCAT-3′-TAMRA
Syncytin 2 env	Forward	5′-GCCTGCAAATAGTCTTCTTT-3′
	Reverse	5′-ATAGGGGCTATTCCCATTAG-3′
	Probe	6FAM- 5′-TGATATCCGCCAGAAACCTCCC-3′-TAMRA
HERV-W env	Forward	5′-CTTCCAGAATTGAAGCTGTAAAGC-3′
	Reverse	5′-GGGTTGTGCAGTTGAGATTTCC-3′
	Probe	6FAM-5′-TTCTTCAAATGGAGCCCCAGATGCAG-3′-TAMRA
TRIM28	Forward	5′-GCCTCTGTGTGAGACCTGTGTAGA-3′
	Reverse	5′-CCAGTAGAGCGCACAGTATGGT-3′
	Probe	6FAM-5′-CGCACCAGCGGGTGAAGTACACC-3′-TAMRA
SETDB1	Forward	5′-GCCGTGACTTCATAGAGGAGTATGT-3′
	Reverse	5′-GCTGGCCACTCTTGAGCAGTA-3′
	Probe	6FAM-5′-TGCCTACCCCAACCGCCCCAT-3′-TAMRA
GAPDH	Forward	5′-CGAGATCCCTCCAAAATCAA-3′
	Reverse	5′-TTCACACCCATGACGAACAT-3′
	Probe	6FAM-5′-TCCAACGCAAAGCAATACATGAAC-3′-TAMRA
